# PrEP use and willingness cascades among GBMSM in 15 Asian countries/territories: an analysis of the PrEP APPEAL survey

**DOI:** 10.1002/jia2.26438

**Published:** 2025-03-28

**Authors:** Gede Benny Setia Wirawan, Heather‐Marie Schimdt, Curtis Chan, Doug Fraser, Jason J. Ong, Michael Cassell, Lei Zhang, Warittha Tieosapjaroen, Nittaya Phanuphak, Weiming Tang, Nicky Suwandi, Kimberly A. Green, Timothy Dobbins, Benjamin R. Bavinton

**Affiliations:** ^1^ Kirby Institute, UNSW Sydney New South Wales Australia; ^2^ Center for Public Health Innovation, Faculty of Medicine Udayana University Denpasar Indonesia; ^3^ Joint United Nations Programme on HIV/AIDS (UNAIDS) Geneva Switzerland; ^4^ World Health Organization Geneva Switzerland; ^5^ School of Translational Medicine Monash University Melbourne Victoria Australia; ^6^ Faculty of Infectious and Tropical Diseases London School of Hygiene and Tropical Medicine London UK; ^7^ FHI 360 Hanoi Vietnam; ^8^ Artificial Intelligence and Modelling in Epidemiology Program, Melbourne Sexual Health Centre Alfred Health Melbourne Victoria Australia; ^9^ China‐Australia Joint Research Center for Infectious Diseases School of Public Health Xi'an Jiaotong University Health Science Center Shaanxi China; ^10^ Institute of HIV Research and Innovation (IHRI) Bangkok Thailand; ^11^ Social Entrepreneurship to Spur Health Global Guangzhou China; ^12^ Institute for Global Health and Infectious Diseases University of North Carolina at Chapel Hill Chapel Hill North Carolina USA; ^13^ Department of Epidemiology University of North Carolina at Chapel Hill Chapel Hill North Carolina USA; ^14^ Asia‐Pacific Coalition on Male Sexual Health Bangkok Thailand; ^15^ PATH, Primary Health Care Seattle Washington USA; ^16^ School of Population Health, UNSW Sydney New South Wales Australia

**Keywords:** men who have sex with men, pre‐exposure prophylaxis, cascade, Asia, HIV, HIV prevention

## Abstract

**Introduction:**

Despite the high HIV incidence among gay, bisexual and other men who have sex with men (GBMSM) and the demonstrated effectiveness of HIV pre‐exposure prophylaxis (PrEP), PrEP is not accessible at scale across Asia. To help inform future scaling efforts, our study aimed to examine PrEP use and willingness to use among GBMSM to identify opportunities and target groups for upscaling PrEP.

**Methods:**

The PrEP APPEAL survey was a cross‐sectional survey, promoted through online advertising and community organizations, from May to November 2022. Eligible participants were adult GBMSM who self‐identified as HIV negative residing in Asia. We constructed two cascades: PrEP use (comprising awareness, lifetime use and current use of PrEP) and PrEP willingness among participants who were aware of PrEP but had never used it (comprising HIV exposure risk, willingness in PrEP and willingness to pay for PrEP). Multivariable logistic regression models identified factors associated with lifetime PrEP use and PrEP willingness.

**Results:**

Of 15,339 participants, 1440 were excluded due to missing data, leaving 13,899 for analysis. Most lived in large or capital cities (68.3%) and in lower‐middle‐income countries (45.1%). The median age was 30 (25−36) years old. For the PrEP use cascade, 82.2% (*n* = 11,427/13,899) of participants were aware of PrEP, 35.0% (*n* = 4000/11,427) had used it before and 70.1% (*n* = 2803/4000) of them were currently on PrEP. For the PrEP willingness cascade, 54.8% of (*n* = 4068/7427) PrEP‐naïve participants engaged in one or more behaviours with a higher risk of HIV acquisition, 73.7% (*n* = 2996/4068) of them expressed willingness to use PrEP and 83.0% (*n* = 2487/2996) of them were willing to pay for PrEP. Multivariable logistic regression models identified system‐level (PrEP availability, accessibility and affordability) predictors of PrEP use. Individual‐level behaviours associated with higher HIV acquisition risks were associated with PrEP use and willingness.

**Conclusions:**

While PrEP uptake was suboptimal, there was high awareness and willingness in PrEP among GBMSM. This is encouraging for future scale‐up efforts. Future PrEP programmes should address system‐level barriers to support PrEP uptake.

## 1 INTRODUCTION

Gay, bisexual and other men who have sex with men (GBMSM) accounted for nearly half (46%) of new HIV cases among adults aged 15–49 years old in the Asia‐Pacific region despite representing a small proportion of the population [1]. According to the Joint United Nations Programme on HIV/AIDS (UNAIDS), the overall prevalence of HIV among GBMSM in the Asia‐Pacific region was 4.7% in 2018–2022 compared to 0.2% in the general adult population [2]. However, the incidence is much higher in some countries in the region, such as the Philippines with a 418% increase in HIV incidence between 2010 and 2023, with large increases among GBMSM [3].

Despite strong evidence of the efficacy and effectiveness of oral pre‐exposure prophylaxis (PrEP) [4, 5, 6], use among those who could benefit most from PrEP has not reached scale in Asia. By 2023, only a handful of Asian countries, such as Thailand and Vietnam, had ongoing national PrEP programmes [7]. Despite increasing PrEP awareness among GBMSM in Asia, this has not necessarily led to increased PrEP use [8]. Barriers remain such as difficulties to access PrEP services. Discrimination and criminalization of GBMSM discourage support for PrEP programmes, whereas stigma against PrEP use is prevalent within the GBMSM community [9, 10, 11]. Costs have also been cited as a barrier to PrEP use [9, 10, 11].

Cascades are useful to evaluate and address critical coverage and equity gaps in HIV prevention programmes, including PrEP [12]. PrEP cascades can assess several stages where improvements can be made in implementation, from eligibility and awareness to willingness to use and uptake, as well as long‐term continuation and adherence [13, 14, 15]. To better understand PrEP use and willingness to use, and to inform future scale‐up programmes in Asian countries/territories, this study aimed to construct cascades of PrEP use and willingness to use, as well as to identify factors associated with both.

## 2 METHODS

### 2.1 Study design and participants

The PrEP APPEAL survey was a cross‐sectional online survey conducted among GBMSM between May and November 2022 in 15 countries and territories in Asia, namely Cambodia, China (excluding Hong Kong and Taiwan), Hong Kong SAR (China), India, Indonesia, Japan, Lao People's Democratic Republic (PDR), Malaysia, Myanmar, Nepal, Philippines, Singapore, Taiwan (China), Thailand and Vietnam. Participants were recruited using paid and unpaid advertising on dating apps and social media platforms as previously described [16]. The survey was open to self‐identifying adult (≥18 years old) GBMSM residing in one of the participating countries/territories who believed themselves to be HIV negative. Explicit electronic consent was given by all participants before they could proceed with the survey. The study was approved by the Human Research Ethics Commission of the University of New South Wales (HC210729). It was submitted to the World Health Organization Ethics Review Committee (ERC.0003690) and was exempted from review.

### 2.2 Variables

We constructed separate cascades for PrEP use and PrEP willingness to allow the analysis of two different indicators in PrEP programme evaluation: uptake and continuation among PrEP users, and willingness to use PrEP among non‐users. The steps of the cascade were drawn from the generic HIV prevention cascade frameworks as described by Moorhouse et al. [17] with the additional step of “higher risk for HIV acquisition” in the PrEP willingness cascade reflecting the priority population who stand to benefit the most from PrEP while recognizing other forms of HIV prevention [6, 18].

The PrEP use cascade consisted of three steps: (1) PrEP awareness (*Have you ever heard of pre‐exposure prophylaxis (“PrEP”)?*); (2) lifetime PrEP use (*Have you ever taken PrEP?*); and (3) current PrEP use (*Are you currently taking PrEP?*). PrEP‐experienced participants who were no longer taking PrEP were asked about their reasons through a single checkbox item allowing for multiple answers. Participants who had heard of PrEP but had never taken PrEP (i.e. PrEP‐naïve) were included in the second cascade of willingness to use PrEP, consisting of three steps: (1) at higher risk for HIV acquisition; (2) willingness to use PrEP (*Would you like to take PrEP but have not?*); and (3) willingness to pay for PrEP. Participants were considered at higher risk for HIV acquisition if they had any of the following in the previous 6 months: more than one sex partner, condomless anal or vaginal sexual intercourse with casual partners, drug use in conjunction with sex (chemsex), injecting drug use, history of receiving payment for sex and history of sexually transmitted infections (STIs) diagnosis [18]. Willingness to pay was initially asked on an ordinal scale (*How much would you be willing to spend per month in total on PrEP, including the medication and any costs associated with clinic visits and tests?*). Participants were dichotomized into willing or unwilling to pay. PrEP‐naïve participants willing to take up PrEP were asked why they had never taken PrEP by selecting applicable answers from a prespecified list.

Covariates included demographic, behavioural and attitudinal items. Country‐level variables included the income level of the country or territory based on World Bank classifications (i.e. lower‐middle income countries [LMICs], upper‐middle‐income countries [UMICs] and high‐income countries [HICs]) and the level of PrEP access and availability in the country or territory at the time of the survey [19]. “Limited” access was defined as places where PrEP is on trial, in post‐trial rollout period or otherwise has limited availability (including Indonesia, Philippines, China, Malaysia, Myanmar, India, Lao PDR, Nepal, Singapore, Hong Kong (China) and Japan), whereas “wider” access was defined as countries where PrEP had been or was in the process of being, integrated to the public health system (Thailand, Vietnam, Cambodia and Taiwan) [16].

Demographic variables included age, gender, sexual identity, education, employment, location of residence and relationship status. The “LGBTQ+ social engagement” scale was generated from two questions regarding the number of friends identifying as LGBTQ+ and the amount of free time spent with those friends (scored 2 to 10), adapted from an existing scale [20].

Attitudinal variables included the willingness to take PrEP to prevent HIV, concern regarding PrEP side effects and comfort in discussing PrEP with healthcare professionals, with response options on a 5‐point Likert scale; this was dichotomized to “agree” (“agree” and “strongly agree” responses) and “disagree” (all other responses).

### 2.3 Analysis

For both PrEP cascades, we report the number and proportion of participants at each cascade step. Bivariable and multivariable logistic regression models were fitted to identify the determinants of lifetime PrEP use among PrEP‐aware participants and willingness to take PrEP among PrEP‐naïve participants at higher risk of HIV acquisition. Analysis for lifetime PrEP use was limited to PrEP‐aware participants, whereas analysis for PrEP willingness was limited to PrEP‐naïve participants at higher risk of HIV acquisition. Variables that were statistically associated (*p*<0.1) with either lifetime PrEP use (PrEP use cascade) or willingness to use PrEP (PrEP willingness cascade) at the bivariable level were block‐entered into the multivariable models. The choice of dependent variables aligns with current paradigms in PrEP programme evaluation [21]. Hosmer‐Lemeshow tests were conducted to assess the goodness of fit with *p*>0.05 as the cut‐off point for a good fit [22]. We report odds ratios (OR), adjusted odds ratios (aOR), 95% confidence intervals (CI) and *p*‐values for these associations. Participants with incomplete responses for any PrEP cascade step variables were excluded from the analysis.

## 3 RESULTS

### 3.1 Characteristics of participants

In total, 15,339 GBMSM participants completed the survey. Participants with missing data on outcome variables (*n* = 1434) were excluded and the final dataset for analysis comprised 13,899 participants. Those excluded from the analysis were more likely to live in lower‐middle‐income countries, (*p* < 0.001), rural regions (*p* < 0.001), have lower education levels (*p* < 0.001), more likely to have engaged in paid sex (*p* = 0.004) and were less likely to identify as a cisgender man (*p* < 0.001).

Table 1 outlines the demographic characteristics of the sample. Nearly half (*n* = 6263, 45%) of the participants resided in LMICs and 33% resided in a country with “wider” access to PrEP (*n* = 4596). The median age was 30 (IQR 25–36). Most participants (*n* = 12,563, 90%) were cisgender men, and 71% (*n* = 9908) identified as gay. Over half of the participants completed an undergraduate or postgraduate degree and 65% (*n* = 9070) were employed full‐time. Most (*n* = 12,486, 90%) participants did not receive any payment for sex in the last 6 months, while 3% (*n* = 396) reported sex work as their primary source of income. Regarding risk behaviours in the last 6 months, 24% (*n* = 3307) reported more than five sexual partners, 54% (*n* = 7537) reported at least one condomless sexual encounter, 18% (*n* = 2453) engaged in chemsex and 6.6% (*n* = 919) injected drugs. Most participants (*n* = 11,320, 81.4%) reported that they had been tested for HIV with a negative result, and the remaining (*n* = 2579, 19%) were never tested but believed themselves to be HIV negative or preferred not to say. Meanwhile, 8% (*n* = 1160) of participants reported having had an STI diagnosis in the last 6 months.

**Table 1:** Demographic characteristics of participants

Variables	*N* = 13,899
Country income group, *n* (%)	
Lower‐middle income	6263 (45)
Upper‐middle income	3318 (24)
High income	4318 (31)
Country PrEP access, *n* (%)	
Partial access	9303 (67)
Wider access	4596 (33)
Location of residence, *n* (%)	
Capital or large city	9498 (68)
Small city or town	3210 (23)
Village or rural area	1191 (9)
Age in years, median (IQR)	30 (25–36)
Age group, *n* (%)	
<20	512 (4)
20−29	6385 (46)
30−39	4728 (34)
40−49	1678 (12)
50−59	515 (4)
≥ 60	81 (0.6)
Gender, *n* (%)	
Cisgender man/male	12563 (90)
Not cisgender man/male	1336 (10)
Sexual orientation, *n* (%)	
Gay	9908 (71)
Bisexual or pansexual	2800 (20)
Straight/heterosexual	272 (2)
Other	919 (7)
Education levels, *n* (%)	
Without high school education	718 (5)
Some high school education	2218 (16)
Vocational certificate	1723 (12)
Undergraduate degree	6561 (47)
Postgraduate degree	2679 (19)
Employment, *n* (%)	
Full‐time	9068 (65)
Part‐time	1421 (10)
Student	1240 (9)
Not working	1774 (13)
Other	396 (3)
Currently in romantic relationship, *n* (%)	
No	8191 (59)
Yes	5708 (41)
LGBTQ+ social engagement, median (IQR)	5 (4–7)
Sex work, *n* (%)	
Not in the last 6 months	12,482 (90)
Sometimes in the last 6 months	1021 (7)
Sex work as the primary income	396 (3)
Number of sexual partners in the last 6 months, *n* (%)	
0−1	4390 (32)
2−5	6203 (45)
> 5	3307 (24)
Condomless intercourse in the last 6 months, *n* (%)	
No	6362 (46)
Yes	7537 (54)
HIV status, *n* (%)	
HIV negative	11,320 (81)
Unknown	2579 (19)
Last HIV test, *n* (%)	
In the last 12 months	9137 (66)
Over 12 months ago	2447 (18)
Never tested	2315 (17)
STI diagnosis in the last 6 months, *n* (%)	
No	12,739 (92)
Yes	1160 (8)
Chemsex in the last 6 months, *n* (%)	
No	11,446 (82)
Yes	2453 (18)
Injected drug use in the last 6 months, *n* (%)	
No	12,980 (93)
Yes	919 (7)
Willing to take PrEP to prevent HIV, *n* (%)	
Disagree	3737 (27)
Agree	10,162 (73)
Worried about PrEP side effects	
Disagree	5930 (43)
Agree	7969 (57)
Comfortable to discuss PrEP with healthcare provider, *n* (%)	
Disagree	5435 (39)
Agree	8464 (61)

### 3.2 PrEP use cascade

**Figure 1:**
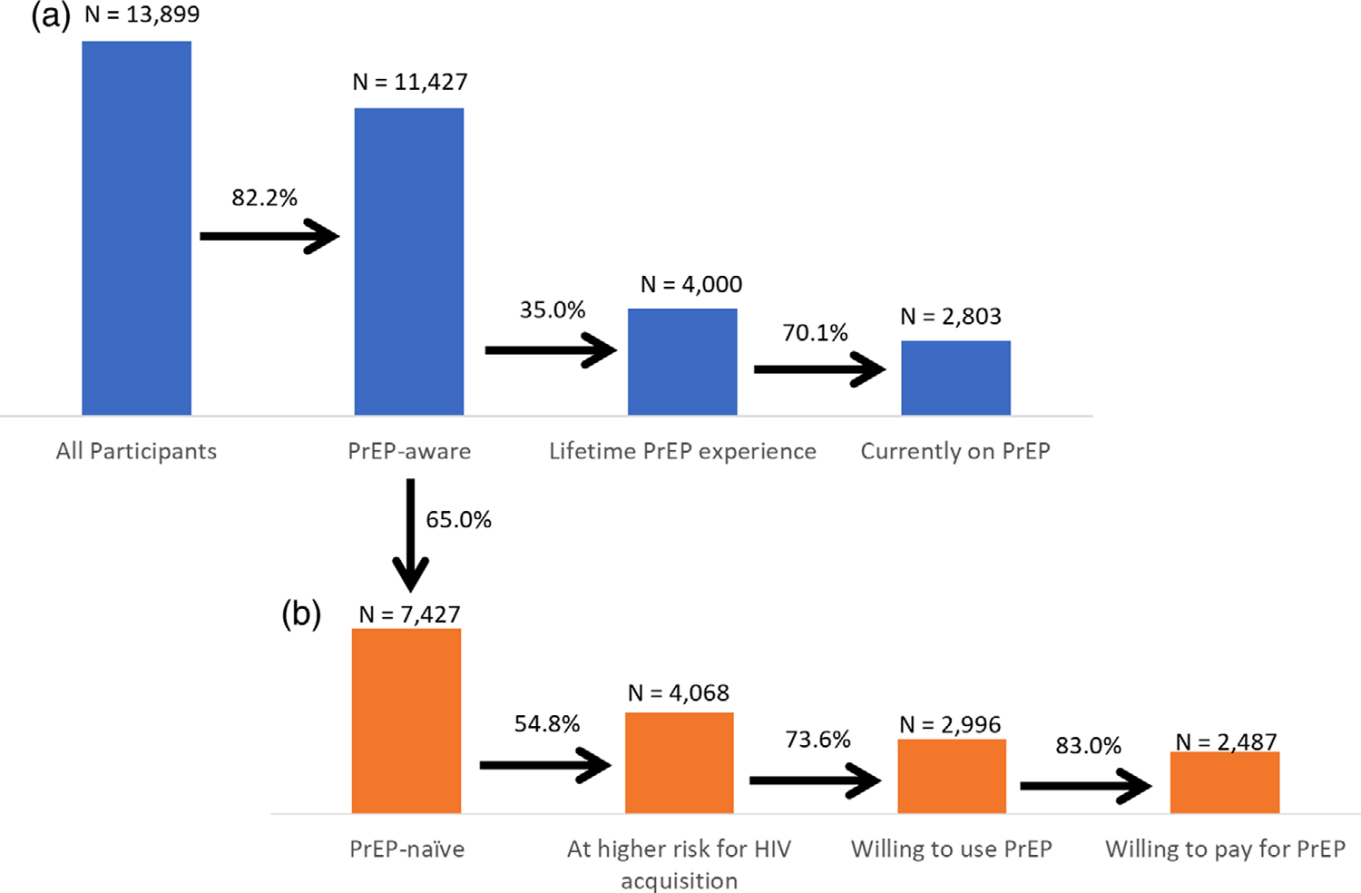
PrEP use and willingness cascades.

Figure 1A shows the PrEP use cascade. Most (*n* = 11,427, 82%) were aware of PrEP. Among PrEP‐aware participants, 4000 (35% of PrEP‐aware participants, 29% of all participants) reported lifetime PrEP use. Among these, 2803 (70.1% of PrEP users, 20% of all participants) were currently taking PrEP, while 26% stated they had stopped PrEP temporarily, and 4% stated they had stopped PrEP permanently. Furthermore, 1413 non‐aware participants (57%, 10% of all participants) engaged in behaviours associated with higher risks of HIV acquisition.

Table 2 describes reasons for stopping PrEP among PrEP‐experienced participants who had discontinued. The three most frequently reported reasons for discontinuing PrEP were not having much sex (*n* = 496, 41%), being too expensive (*n* = 327, 27%) and being concerned about side effects (*n* = 310, 26%). Other reasons related to system‐level barriers included PrEP no longer being available where participants lived (12%), participants not knowing where to get PrEP (11%) and PrEP becoming too inconvenient for participants (8%).

**Table 2:** Reasons for stopping PrEP or not taking up PrEP

Reasons	Stopped using PrEP^a^ *n* = 1197	Never tried PrEP^b^ *n* = 2996
I am not having much sex	496 (41%)	608 (20%)
Too expensive	327 (27%)	1286 (43%)
I am concerned about the side effects	310 (26%)	989 (33%)
I do not like taking pills regularly	240 (20%)	447 (15%)
I prefer to use condoms	206 (17%)	370 (12%)
I got into a monogamous relationship	139 (12%)	112 (4%)
Not available where I live	138 (12%)	517 (17%)
Do not know where to get PrEP	134 (11%)	1727 (58%)
I am not at high risk of HIV	131 (11%)	164 (6%)
Too inconvenient	97 (8%)	238 (8%)
Cannot get prescription	95 (8%)	559 (19%)
COVID‐19 made it too hard to get	80 (7%)	118 (4%)
I am concerned about what my friends and family would think of me	55 (5%)	364 (12%)
My sexual partner(s) did not like me taking PrEP	12 (1%)	14 (1%)
I was denied access to PrEP by a healthcare provider	11 (1%)	36 (1%)

*Note*: Percentages do not add up to 100% as participants were able to select more than one reason.

^a^ Among participations with lifetime experience using pre‐exposure prophylaxis (PrEP).

^b^ Among participants without prior history of using pre‐exposure prophylaxis (PrEP).

### 3.3 PrEP willingness cascade

The PrEP willingness cascade started with PrEP‐naïve participants (*n* = 7431) as shown in Figure 1B. Among these, 4068 (55%) engaged in practices associated with higher risks of HIV acquisition. Among PrEP‐naïve participants engaged in practices associated with higher risks of HIV acquisition, 2996 (74% of PrEP‐naïve participants at higher risk, 69% of all PrEP‐naïve participants) were willing to use PrEP. Of these, 2487 (83% of PrEP‐naïve participants at higher risk, 57% of all PrEP‐naïve participants and 31% of the total sample) were willing to pay for PrEP.

Table 2 shows non‐mutually exclusive reasons for not being on PrEP among PrEP‐naïve participants who were willing to use PrEP and at higher risk of HIV acquisition. The two most frequently cited reasons for never taking up PrEP were not knowing where to get PrEP (*n* = 1727, 58%) and it being too expensive (*n* = 1286, 43%). Other reasons related to system‐level barriers included PrEP not being available where participants lived (17%), being unable to get prescriptions (19%) and perceiving PrEP as too inconvenient for participants (8%).

### 3.4 Factors associated with PrEP uptake

The multivariable regression model shown in Table 3 was a good fit to the data with Hosmer‐Lemeshow *p* = 0.534. In the multivariable model, variables positively associated with lifetime PrEP use were: wider country‐level PrEP access, older age, non‐cisgender male identity, employment, being in a romantic relationship, sex work, a higher number of sexual partners, having had condomless sex, STI diagnosis, chemsex, or injecting drug use in the last 6 months, expressed willingness to use PrEP to prevent HIV and comfort in discussing PrEP with healthcare providers. Variables negatively associated with lifetime PrEP use were: living in an upper‐middle or high‐income country, non‐gay sexual identity, having a university degree, being of unknown HIV status, having their last HIV test over a year ago and expressing concern about PrEP side effects. Salient factors based on group distribution and effect sizes included participants living in higher‐income countries compared to lower‐middle‐income countries (aOR 0.53, 95% CI 0.47–0.60), living in countries with wider PrEP access (aOR 2.11, 95% CI 1.92–2.33), having five or more sex partners in the last 6 months compared to those having 0–1 partners (aOR 2.15, 95% CI 1.88–2.46), history of chemsex in the last 6 months (aOR 1.95, 95% CI 1.73–2.18) and agreeing or strongly agreeing with a statement expressing concern about PrEP side effects (aOR 0.64, 95% CI 0.58–0.70). 4

**Table 3:** Logistic regression for factors associated with lifetime PrEP use

	PrEP‐experienced, *n* (%)		Bivariable regression		Multivariable regression	
Variables *N* = 11,427	No (*N* = 7427)	Yes (*N* = 4000)	OR (95% CI)	*p*‐value	aOR (95% CI)	*p*‐value
Country groups						
Lower‐middle income	2782 (37.3)	1867 (46.7)	Ref.	<0.001	Ref.	
Upper‐middle income	1915 (30.0)	952 (23.8)	0.74 (0.67–0.82)		0.73 (0.65–0.83)	
High income	2730 (32.7)	1181 (29.5)	0.65 (0.59–0.71)		0.53 (0.47–0.60)	
PrEP access by country						<0.001
Partial access	5210 (70.2)	2093 (52.3)	Ref.	<0.001	Ref.	
Wider access	2217 (29.8)	1907 (47.7)	2.14 (1.98–2.32)		2.11 (1.92–2.33)	
Residence						0.078
Town, villages or rural areas	2248 (30.3)	1078 (26.9)	Ref.	<0.001	Ref.	
Capital or large cities	5179 (69.7)	2922 (73.1)	1.18 (1.08–1.28)		1.10 (0.99–1.22)	
Age groups						
<20	279 (3.8)	83 (2.1)	Ref.	<0.001	Ref.	
20−29	3340 (45.0)	1836 (45.9)	1.85 (1.44–2.37)		1.43 (1.05–1.96)	
30−39	2528 (34.0)	1459 (36.5)	1.94 (1.51–2.50)		1.62 (1.17–2.23)	
40−49	939 (12.6)	466 (11.7)	1.67 (1.27–2.18)		1.82 (1.29–2.55)	
50−59	292 (3.9)	141 (3.5)	1.62 (1.18–2.23)		2.91 (1.96–4.32)	
≥ 60	49 (0.7)	15 (0.4)	1.03 (0.55–1.93)		1.72 (0.82–3.58)	
Gender						<0.001
Cisgender man/male	6950 (93.6)	3526 (88.1)	Ref.	<0.001	Ref.	
Not cisgender man/male	477 (6.5)	474 (11.9)	1.95 (1.71–2.22)		1.56 (1.31–1.85)	
Sexual orientation						<0.001
Gay	5425 (73.0)	3170 (79.2)	Ref.	<0.001	Ref.	
Not gay	2002 (27.0)	830 (20.8)	0.71 (0.65–0.78)		0.73 (0.65–0.81)	
Education levels						0.028
Without university degree	1232 (16.6)	897 (22.4)	Ref.	<0.001	Ref.	
University degree	6195 (83.4)	3103 (77.6)	0.69 (0.63–0.75)		0.87 (0.76–0.98)	
Employment						0.001
Not employed	1765 (23.8)	813 (20.3)	Ref.	<0.001	Ref.	
Employed	5662 (76.2)	3187 (79.7)	1.22 (1.11–1.34)		1.21 (1.08–1.37)	
Relationship						0.001
No	4590 (61.8)	2066 (51.6)	Ref.	<0.001	Ref.	
Yes	2837 (38.2)	1934 (48.4)	1.51 (1.40–1.63)		1.27 (1.08–1.37)	
Social engagement in the LGBTQ+ community (2−10), each incremental score, median (IQR)	5 (4−6)	6 (5−7)	1.15 (1.12–1.18)	<0.001	1.01 (0.98–1.04)	0.702
Sex work						
Not in the last 6 months	6912 (93.1)	3427 (85.7)	Ref.	<0.001	Ref.	
Sometimes in the last 6 months	422 (5.7)	358 (9.0)	1.71 (1.47–1.98)		1.23 (1.03–1.47	
Sex work is primary income	93 (1.2)	215 (5.3)	4.66 (3.64–.597)		1.80 (1.33–2.44)	
Number of partners in the last 6 months						
0−1	2605 (35.1)	744 (18.6)	Ref.	<0.001	Ref.	
2−5	3454 (46.5)	1752 (43.8)	1.78 (1.61–1.96)		1.27 (1.14–1.43)	
> 5	1368 (18.4)	1505 (37.6)	3.85 (3.46–4.30)		2.15 (1.88–2.46)	
HIV status						<0.001
HIV negative	5999 (80.8)	3808 (95.2)	Ref.	<0.001	Ref.	
Unknown	1428 (19.2)	192 (4.8)	0.21 (0.18–0.25)		0.63 (0.50–0.80)	
Last HIV test						<0.001
In the last 12 months	4444 (59.9)	3656 (91.4)	Ref.	<0.001	Ref.	
Over 12 months ago	1664 (22.4)	281 (7.0)	0.21 (0.18–0.23)		0.25 (0.22–0.29)	
Never tested	1319 (17.7)	63 (1.6)	0.06 (0.05–0.08)		0.11 (0.07–0.15)	
Condomless sex in the last 6 months						<0.001
No	3914 (52.7)	1258 (31.5)	Ref.	<0.001	Ref.	
Yes	3513 (47.3)	2742 (68.6)	2.12 (1.95–2.32)		1.65 (1.50–1.82)	
STI diagnosis in the last 6 months						<0.001
No	7004 (94.3)	3381 (84.5)	Ref.	<0.001	Ref.	
Yes	423 (5.7)	619 (15.5)	3.03 (2.66–3.45)		1.54 (1.32–1.79)	
Chemsex in the last 6 months						<0.001
No	6553 (88.2)	2740 (68.5)	Ref.	< 0.001	Ref.	
Yes	874 (11.8)	1260 (31.5)	3.45 (3.13–3.80)		1.95 (1.73–2.18)	
Injected drug use in the last 6 months				<0.001		<0.001
No	7090 (95.5)	3528 (88.2)	Ref.			
Yes	337 (4.5)	472 (11.8)	2.82 (2.43–3.26)			
Attitude: Willing to take PrEP to prevent HIV						<0.001
Disagree	1918 (25.8)	767 (19.2)	Ref.	<0.001	Ref.	
Agree and strongly agree	5509 (74.2)	3233 (80.8)	1.47 (1.33–1.61)		1.32 (1.18–1.49)	
Attitude: Worried about PrEP side effects						<0.001
Disagree	2796 (37.6)	1980 (49.5)	Ref.	<0.001	Ref.	
Agree and strongly agree	4631 (62.4)	2020 (50.5)	0.62 (0.57–0.67)		0.64 (0.58–0.70)	
Attitude: Comfortable to discuss PrEP with healthcare provider						<0.001
Disagree	3153 (42.4)	1095 (27.4)	Ref.	<0.001	Ref.	
Agree and strongly agree	4274 (57.6)	2905 (72.6)	1.96 (1.80–2.12)		1.57 (1.42–1.74)	

*Note*: This table is limited to PrEP‐aware participants, hence *N* = 11,427.

### 3.5 Factors associated with PrEP willingness

The multivariable regression model shown in Table 4 was a good fit to the data with Hosmer‐Lemeshow *p* = 0.646. Factors positively associated with PrEP willingness were having a university degree, sex work, higher number of sex partners, STI diagnosis history and willingness to use PrEP to prevent HIV. Living in an upper‐middle income country (compared to LMICs), chemsex, expressed concern for PrEP side effects and comfort discussing PrEP with a healthcare provider were negatively associated with PrEP willingness. Salient factors included participants living in UMICs compared to LMICs (aOR 0.6, 95% CI 0.53–0.79), having five or more partners in the last 6 months compared to those having 0–1 partners (aOR 2.34, 95% CI 1.87–2.92) and agreeing or strongly agreeing to statements expressing willingness to use PrEP to prevent HIV (aOR 3.98, 95% CI 3.36–4.71), concerns about side effects (aOR 0.76, 95% CI 0.65–0.89) and comfort discussing PrEP with healthcare providers (aOR 0.76, 95% CI 0.71–0.97).

**Table 4:** Logistic regression for factors associated with PrEP willingness

	Willingness in PrEP, *n* (%)		Bivariable regression		Multivariable regression	
Variables *N* = 3901	No (*N* = 1072)	Yes (*N* = 2997)	OR (95% CI)	*p*‐value	aOR (95% CI)	*p*‐value
Country groups				<0.001		<0.001
Lower‐middle income	365 (34.1)	1134 (37.9)	Ref.		Ref.	
Upper‐middle income	325 (30.3)	688 (23.0)	0.68 (0.57–0.81)		0.65 (0.53–0.79)	
High income	382 (35.6)	1174 (39.1)	0.99 (0.84–1.17)		0.89 (0.74–1.08)	
PrEP access by country				0.310		
Partial access	705 (65.8)	2021 (67.5)	Ref.			
Wider access	367 (34.2)	975 (32.5)	0.93 (0.80–1.07)			
Residence				0.671		
Town, villages or rural areas	338 (31.5)	924 (30.8)	Ref.			
Capital or large cities	734 (67.5)	2073 (69.2)	1.03 (0.89–1.20)			
Age groups				0.072		0.286
<20	34 (3.2)	121 (4.0)	Ref.		Ref.	
20−29	485 (45.2)	1386 (46.3)	0.80 (0.54–1.20)		0.73 (0.47–1.12)	
30−39	365 (34.1)	1003 (33.5)	0.72 (0.52–1.15)		0.69 (0.45–1.07)	
40−49	129 (12.0)	368 (12.3)	0.80 (0.52–1.23)		0.71 (0.45–1.14)	
50−59	35 (4.2)	104 (3.5)	0.65 (0.39–1.09)		0.64 (0.36–1.11)	
≥ 60	14 (1.3)	14 (0.5)	0.28 (0.12–0.65)		0.35 (0.14–0.86)	
Gender				0.087		0.182
Cisgender man/male	991 (92.4)	2816 (94.0)	Ref.		Ref.	
Not cisgender man/male	81 (7.6)	180 (6.0)	0.79 (0.60–1.03)		0.20 (0.61–1.10)	
Sexual orientation				0.424		
Gay	773 (72.1)	2198 (73.4)	Ref.			
Not gay	299 (27.9)	798 (26.6)	0.94 (0.80–1.10)			
Education levels				0.002		0.003
Without university degree	240 (22.4)	537 (17.9)	Ref.		Ref.	
University degree	832 (77.6)	2459 (82.1)	1.32 (1.11–1.57)		1.34 (1.11–1.63)	
Employment				0.803		
Not employed	246 (23.0)	699 (23.3)	Ref.			
Employed	826 (77.0)	2297 (76.7)	0.98 (0.83–1.16)			
Relationship				0.011		0.245
No	616 (57.5)	1855 (61.9)	Ref.		Ref.	
Yes	456 (42.5)	1141 (38.1)	0.83 (0.72–0.96)		0.91 (0.78–1.06)	
Social engagement in the LGBTQ+ community (2−10), each incremental score	5 (4–6)	6 (4–7)	1.05 (1.01–1.11)	0.016	1.02 (0.97–1.07)	0.449
Sex work				<0.001		<0.001
Sex work as primary income	33 (3.1)	42 (1.4)	Ref.		Ref.	
Sometimes in the last 6 months	62 (5.8)	239 (8.0)	3.03 (1.77–5.17)		3.02 (1.71–5.34)	
Not in the last 6 months	977 (91.1)	2715 (90.6)	2.18 (1.38–3.47)		2.62 (1.58–4.35)	
Number of partners in the last 6 months				<0.001		<0.001
0−1	379 (35.4)	599 (20.0)	Ref.		Ref.	
2−5	492 (45.9)	1598 (53.3)	2.05 (1.74–2.42)		1.89 (1.59–2.26)	
> 5	201 (18.7)	799 (26.7)	2.51 (2.05–3.07)		2.34 (1.87–2.92)	
HIV status				0.635		
HIV negative	878 (81.9)	2434 (81.3)	Ref.			
Unknown	194 (18.1)	572 (18.7)	1.04 (0.87–1.25)			
Last HIV test						
In the last 12 months	708 (66.0)	1902 (63.5)	Ref.	0.171		
Over 12 months ago	215 (20.1)	609 (20.3)	1.05 (0.88–1.26)			
Never tested	149 (13.9)	485 (16.2)	1.21 (0.99–1.48)			
Condomless sex in the last 6 months				<0.001		0.238
No	179 (16.7)	376 (12.6)	Ref.		Ref.	
Yes	893 (83.3)	2620 (87.4)	1.40 (1.15–1.70)		1.16 (0.91–1.47)	
STI diagnosis in the last 6 months				0.002		0.009
No	986 (92.0)	2659 (88.8)	Ref.		Ref.	
Yes	86 (8.0)	337 (11.2)	1.45 (1.13–1.86)		1.44 (1.10–1.88)	
Chemsex in the last 6 months				0.002		0.031
No	805 (75.1)	2389 (79.8)	Ref.		Ref.	
Yes	267 (24.9)	607 (20.3)	0.77 (0.65–0.90)		0.81 (0.67–0.98)	
Injected drug use in the last 6 months				0.029		0.342
No	966 (90.1)	2765 (92.3)	Ref.		Ref.	
Yes	106 (9.9)	231 (7.7)	0.76 (0.60–0.97)		0.87 (0.66–1.15)	
Attitude: Willing to take PrEP to prevent HIV				<0.001		<0.001
Disagree	460 (42.9)	517 (17.3)	Ref.		Ref.	
Agree and strongly agree	612 (57.1)	2479 (82.7)	3.61 (3.09–4.20)		3.98 (3.36–4.71)	
Attitude: Worried about PrEP side effects				0.082		0.001
Disagree	405 (37.8)	1223 (40.8)	Ref.		Ref.	
Agree and strongly agree	667 (62.2)	1773 (59.2)	0.88 (0.76–1.02)		0.76 (0.65–0.89)	
Attitude: Comfortable to discuss PrEP with healthcare provider				0.009		0.021
Disagree	497 (46.4)	1252 (41.8)	Ref.		Ref.	
Agree and strongly agree	575 (53.6)	1744 (58.2)	1.20 (1.05–1.39)		0.76 (0.71–0.97)	

*Note*: This table is limited to PrEP‐naïve participants who engaged in one or more behaviours associated with higher risks of HIV acquisition, hence *N* = 3901.

## 4 DISCUSSION

Our analysis included 13,899 participants from 15 Asian countries/territories, which is to our knowledge the largest study of its kind in this region. While comparison is hard to come by for the scale of our study, the demographics of participants based on age, education and employment were comparable with recently conducted national‐level surveys of GBMSM [23, 24, 25, 26, 27]. We constructed PrEP use and PrEP willingness cascades, which identified high PrEP awareness among participants (82%), with one‐third of lifetime PrEP uptake (35%) among PrEP‐aware participants. We found a high level of PrEP willingness (74%) among PrEP‐naïve participants at higher risk and a high willingness to pay for PrEP (83%) among willing participants. Uptake and willingness to use PrEP were the highest in LMICs compared to other country income groups. PrEP use was higher in countries with wider access to PrEP. PrEP use and willingness were higher among participants reporting more sex partners or any STI diagnosis in the past 6 months.

The 35% lifetime PrEP use we found is comparable with previous country‐level estimates of PrEP use, which varied between 2% and 35% among GBMSM in Indonesia, Malaysia, China and Thailand between 2017 and 2020 [15, 28, 29, 30]. However, the level of awareness (82%) we found was significantly higher compared to that previously reported in a 2022 meta‐analysis which stood at 37% (95% CI 31−43%) in the Western Pacific and 18% (95% CI 13−23%) in the Southeast Asia region. While not as significant, PrEP willingness in our study among all PrEP‐naïve participants (69%) was higher compared to findings in the same meta‐analysis which were 57% (95% CI 48−65%) and 61% (48−74%) in the Western Pacific and the Southeast Asia regions, respectively [8]. As PrEP awareness has recently grown following implementation and scale‐up in several settings, our higher estimates of awareness and willingness to use PrEP may represent the global trend among GBMSM [8, 31].

High PrEP willingness among PrEP‐naïve participants at higher risks of HIV acquisition indicates an urgent need for greater PrEP availability and access in this region. Providing PrEP to those who can most benefit from its use and are already willing to take it can be the largest potential gain in controlling the HIV epidemic. Our data showed PrEP willingness is associated with behaviours linked to a higher risk of HIV transmission consistently across countries with different PrEP availability (Tables S3 and S4), further showcasing the opportunity for PrEP scale‐up. Failure to provide would be a missed opportunity for global PrEP implementation. PrEP and PrEP services should be available, accessible and acceptable to those who can benefit the most from its use. Further efforts from governments, clinical bodies and international organizations are needed to reduce barriers to PrEP access among those at higher risk of HIV acquisition. However, our data suggest there are availability, accessibility and affordability barriers to PrEP uptake and continued use.

PrEP availability was a significant factor demonstrated through higher PrEP use in PrEP‐accessible countries, and a perceived lack of availability was a common reason to discontinue or never use PrEP (Table 2). In a 2019 Asia‐Pacific regional consultation on PrEP, healthcare providers, programme implementers, government officials and key population representatives from Indonesia, Cambodia, Myanmar and Sri Lanka cited lack of country‐specific evidence contributed to the slow rollout of PrEP [32]. There is also resistance stemming from “conservative or homophobic attitudes” from the communities, society at large, and (crucially) within Ministries of Health in the region impeding the generation of aforementioned country‐specific evidence and slowing the rollout process in general [32]. A case study of Indonesia showed stigma and discrimination is a deep‐seated problem where most instances of discriminatory practices occur at the local level by local policymakers, implementers and even healthcare providers [33]. A fundamental cultural shift and normalization is required to overcome this problem; hopefully realized through a continuous process of advocacy by academics, local medical and GBMSM communities, human rights organizations and international health organizations [34].

Second, the processes to acquire PrEP may be inaccessible even when PrEP is theoretically available. Common reasons participants for not initiating PrEP or PrEP discontinuation included difficulties in obtaining prescriptions, PrEP being too inconvenient or participants simply not knowing where to get PrEP. Similarly, comfort in discussing PrEP was a significant factor associated with lifetime PrEP use, especially in countries with wider PrEP availability (Table S3). The salience of these reasons persists after removing participants with non‐barrier‐related (e.g. monogamous relationship, not having much sex) or preference‐related (e.g. do not like pills, prefer condoms) reasons for unwillingness to use PrEP (Table S2). These reasons relate to aspects of the accessibility framework, including the ability of potential PrEP users to perceive service availability, freely seek the service, physically reach the service, and find the service appropriate for their needs and values [35]. Enduring accessibility barriers can at least be partially attributed to the under‐utilization of differentiated service delivery options for PrEP, resulting in a reliance on inflexible and potentially expensive medicalized models. Examples of community‐friendly models that empower nurses, pharmacists and community health workers to provide PrEP have been shown to be effective in various settings [32, 36, 37]. To address accessibility issues, other contexts should implement these alternative de‐medicalized PrEP provision models.

Lastly, affordability was an important factor in PrEP use shown by the salience of expensive costs as reasons for unwillingness to use PrEP and its discontinuation. In some Asian countries, PrEP has been provided for free or at a heavily subsidized price, enabled by external donor funding typically directed towards lower‐middle‐income countries [35]. However, this model faces major challenges when funding ceases or the donor‐funded programme transitions to the national health system [35, 38, 39]. Allocating domestic financing towards PrEP can be challenging in many countries, including HICs and middle‐income countries not receive external donor funding [40]. Without subsidy, PrEP can be unaffordable for many people, limiting its public health potential. The amount people are willing to pay for PrEP is low compared to its actual market price. In Indonesia, for example, PrEP services were estimated to cost USD 365 per user per year while most participants were only willing to pay up to USD 15 per month [16, 41]. We also found that 27% of participants no longer on PrEP reported cost as a reason. Similarly, 43% of willing PrEP‐aware participants did not initiate PrEP due to cost.

Additional efforts are also needed to promote awareness and willingness to use PrEP (i.e. demand generation). Awareness does not always lead to demand. PrEP awareness increased from 18%–37% in the 2022 meta‐analysis [8] to 82% found in our data, while PrEP willingness only rose from 57%–61% to 69%. There were also 20% of PrEP‐aware participants who were neither PrEP‐experienced nor willing to use PrEP, a significant proportion among them reported behaviours with a higher risk of HIV acquisition, such as having condomless sex (38%) and having more than one partner (53%). This could be a useful target for PrEP awareness campaigns. Gain‐framed messaging has been proven effective in demand‐generation efforts, focusing on what PrEP can give users (e.g. protection, freedom, peace of mind) [42]. Other than availability, accessibility and affordability barriers discussed above, attitudes such as perceived risk of side effects, effectiveness, social norms and convenience also contribute to this phenomenon [8]. Moving forward, demand generation efforts must go beyond only increasing PrEP awareness and should also focus on willingness to use PrEP among both potential PrEP users by addressing barriers both real and perceived.

These results should be considered in light of some limitations. Our study utilized convenience online sampling with its associated risks of sampling bias. Compared to their population size and total sample size, some countries/territories were over‐represented, while others were under‐represented especially in regards to internet use required to participate in the study [16]. However, these limitations are expected considering the difficulty of conducting probability sampling in socially marginalized populations such as GBMSM. It was also mitigated by our use of a multi‐pronged recruitment strategy, utilizing community groups, community influencers, as well as paid advertisements on social media and dating applications [16].

We also encourage a careful interpretation of the potential bias from linking the recent risk behaviours and lifetime PrEP use. However, such bias risks were minimized in our context considering PrEP scale‐up (and, therefore, the first instances of PrEP use) was a recent phenomenon in most of the included countries/territories at the time of data collection (mid‐2023) leading to potential overlaps between periods of recent risk behaviours and first instances of PrEP use. As these data were self‐reported, it is also subject to risks of social desirability and recall biases regarding sexual behaviour, PrEP attitudes and other variables.

## 5 CONCLUSIONS

Across Asia, we found high levels of PrEP awareness and willingness. While encouraging, in the context of comparatively lower levels of PrEP use, high PrEP awareness shows an urgent need to remove barriers to PrEP access. Indeed, addressing system‐level barriers including availability, accessibility and affordability, for example through the adoption of differentiated service delivery models, could help countries to rapidly achieve population‐level HIV incidence reductions. Additionally, demand generation focussing on willingness to use PrEP on top of mere awareness could be particularly important for individuals who could benefit from this HIV prevention option, but who may not currently be aware of or willing to take up PrEP.

## COMPETING INTERESTS

BRB and NP have received research funding, travel and honoraria from ViiV Healthcare and Gilead Sciences. GBSW, CC, JJO, DO and H‐MS have no competing interests.

## AUTHORS’ CONTRIBUTIONS

BRB, H‐MS, JJO, KAG, NP, MC and NS conceptualized and designed the research study. H‐MS, CC, DF, JJO, MC, LZ, WT, NP, NS, KAG and BRB collected the data. GBSW analysed the data with support from BRB and TD and wrote the initial draft. All authors reviewed, revised and approved the manuscript.

## FUNDING

This study was supported by funding from the World Health Organization, the Kirby Institute and the Outstanding Young Scholars Support Program. The Australian arm of the study was supported by funds from ViiV Healthcare, the NSW Ministry of Health, MAC AIDS Fund and the Australian Government Department of Health. GBSW also acknowledges and thanks the postgraduate scholarship provided by the Indonesian Endowment Funds for Education for providing the opportunity to be involved in this project.

## Supporting information




**Table S1.1**. PrEP use cascade by country.
**Table S1.2**. PrEP willingness cascade by country.

**Table S2**. Subgroup analysis for reasons for stopping PrEP or not taking up PrEP based on being in a monogamous relationship as a reason for not being on PrEP.

**Table S3**. Stratified logistic regression for factors associated with lifetime PrEP use.

**Table S4**. Stratified logistic regression for factors associated with PrEP willingness among PrEP‐naïve participants with higher risk of HIV.

## Data Availability

A de‐identified copy of the dataset and syntax used in the analysis is available from the authors, upon request.

## References

[1] UNAIDS . UNAIDS Data Book 2022. Geneva: UNAIDS; 2023.

[2] UNAIDS . Asia‐Pacific Regional Factsheet 2023. Geneva: UNAIDS; 2023.

[3] UNAIDS . Asia Pacific Country HIV Snapshots 2023. 2023. Accessed 30 October 2023. https://www.aidsdatahub.org/resource/asia‐pacific‐country‐hiv‐snapshots‐2023

[4] Spinner CD , Boesecke C , Zink A , Jessen H , Stellbrink H‐J , Rockstroh JK , et al. HIV pre‐exposure prophylaxis (PrEP): a review of current knowledge of oral systemic HIV PrEP in humans. Infection. 2016;44:151–158. 10.1007/s15010-015-0850-2 26471511

[5] Fonner VA , Dalglish SL , Kennedy CE , Baggaley R , O'Reilly KR , Koechlin FM , et al. Effectiveness and safety of oral HIV preexposure prophylaxis for all populations. AIDS. 2016;30:1973–1983. 10.1097/QAD.0000000000001145 27149090 PMC4949005

[6] Grulich AE , Guy R , Amin J , Jin F , Selvey C , Holden Jo , et al. Population‐level effectiveness of rapid, targeted, high‐coverage roll‐out of HIV pre‐exposure prophylaxis in men who have sex with men: the EPIC‐NSW prospective cohort study. Lancet HIV. 2018;5:e629–e637. 10.1016/S2352-3018(18)30215-7 30343026

[7] PrEP Watch . 2024 Q1 Global PrEP Tracker. 2024. Accessed 26 June 2023. https://www.prepwatch.org/resources/global‐prep‐tracker/

[8] Sun Z , Gu Q , Dai Y , Zou H , Agins B , Chen Q , et al. Increasing awareness of HIV pre‐exposure prophylaxis (PrEP) and willingness to use HIV PrEP among men who have sex with men: a systematic review and meta‐analysis of global data. J Int AIDS Soc. 2022;25: e25883. 10.1002/jia2.25883 35255193 PMC8901150

[9] Nguyen LH , Nguyen HLT , Tran BX , Larsson M , Rocha LEC , Thorson A , et al. A qualitative assessment in acceptability and barriers to use pre‐exposure prophylaxis (PrEP) among men who have sex with men: implications for service delivery in Vietnam. BMC Infect Dis. 2021;21:472. 10.1186/s12879-021-06178-5 34030652 PMC8147440

[10] Rosen AO , Wickersham JA , Altice FL , Khati A , Azwa I , Tee V , et al. Barriers and facilitators to pre‐exposure prophylaxis by men who have sex with men and community stakeholders in Malaysia. Int J Environ Res Public Health. 2023;20(9): 5669. 10.3390/ijerph20095669 37174187 PMC10177799

[11] Philbin MM , Hirsch JS , Wilson PA , Ly AnT , Giang LeM , Parker RG . Structural barriers to HIV prevention among men who have sex with men (MSM) in Vietnam: diversity, stigma, and healthcare access. PLoS One. 2018;13:e0195000. 10.1371/journal.pone.0195000 29614104 PMC5882136

[12] Haber N , Pillay D , Porter K , Bärnighausen T . Constructing the cascade of HIV care: methods for measurement. Curr Opin HIV AIDS. 2016;11:102–108. 10.1097/COH.0000000000000212 26545266

[13] Parsons JT , Rendina HJ , Lassiter JM , Whitfield THF , Starks TJ , Grov C . Uptake of HIV pre‐exposure prophylaxis (PrEP) in a national cohort of gay and bisexual men in the United States. J Acquir Immune Defic Syndr. 2017;74:285–292. 10.1097/QAI.0000000000001251 28187084 PMC5315535

[14] Schaefer R , Gregson S , Fearon E , Hensen B , Hallett TB , Hargreaves JR . HIV prevention cascades: a unifying framework to replicate the successes of treatment cascades. Lancet HIV. 2019;6:e60–e66. 10.1016/S2352-3018(18)30327-8 32066995 PMC7025885

[15] Cempaka R , Wardhani B , Sawitri AAS , Januraga PP , Bavinton B . PrEP use awareness and interest cascade among MSM and transgender women living in Bali, Indonesia. Trop Med Infect Dis. 2020;5:158. 10.3390/tropicalmed5040158 33050477 PMC7709693

[16] Chan C , Fraser D , Schmidt H , Green K , Cassel M , Ong J , et al. PrEP product awareness, preferences, and past experiences among transgender women and men who have sex with men in Asia and Australia: the PrEP APPEAL study report. Sydney: Kirby Institute; 2023.

[17] Moorhouse L , Schaefer R , Thomas R , Nyamukapa C , Skovdal M , Hallett TB , et al. Application of the HIV prevention cascade to identify, develop and evaluate interventions to improve use of prevention methods: examples from a study in east Zimbabwe. J Int AIDS Soc. 2019;22(Suppl 4):e25309. 10.1002/jia2.25309 31328375 PMC6643077

[18] Holt M , Broady TR , Mao L , Chan C , Rule J , Ellard J , et al. Increasing preexposure prophylaxis use and ‘net prevention coverage’ in behavioural surveillance of Australian gay and bisexual men. AIDS. 2021;35:835–840. 10.1097/QAD.0000000000002797 33587442

[19] World Bank . World Bank Country and Lending Groups. 2023. Accessed 19 June 2023. https://datahelpdesk.worldbank.org/knowledgebase/articles/906519‐world‐bank‐country‐and‐lending‐groups

[20] Chan C , Bavinton BR , Prestage GE , Broady TR , Mao L , Rule J , et al. Changing levels of social engagement with gay men is associated with HIV related outcomes and behaviors: trends in Australian Behavioral Surveillance 1998–2020. Arch Sex Behav. 2022;51:2509–2521. 10.1007/s10508-022-02310-x 35672592 PMC9293873

[21] Dunbar MS , Kripke K , Haberer J , Castor D , Dalal S , Mukoma W , et al. Understanding and measuring uptake and coverage of oral pre‐exposure prophylaxis delivery among adolescent girls and young women in sub‐Saharan Africa. Sex Health. 2018;15:513–521. 10.1071/SH18061 30408431 PMC6429961

[22] Bewick V , Cheek L , Ball J . Statistics review 14: logistic regression. Crit Care. 2005;9:112–118. 10.1186/cc3045 15693993 PMC1065119

[23] Li C‐W , Ku SW‐W , Huang P , Chen L‐Yu , Wei H‐T , Strong C , et al. Factors associated with methamphetamine dependency among men who have sex with men engaging in chemsex: findings from the COMeT study in Taiwan. Int J Drug Policy. 2021;93:103119. 10.1016/j.drugpo.2021.103119 33468444

[24] Ditangco R , Mationg ML . HIV incidence among men who have sex with men (MSM) in Metro Manila, the Philippines: a prospective cohort study 2014–2018. Medicine (Baltimore). 2022;101:e30057. 10.1097/MD.0000000000030057 36107537 PMC9439796

[25] Yang X , Kang W , Zhang Z , Tang H , Zhang D , Sun L , et al. HIV pre‐exposure prophylaxis cascade stages among men who have sex with men with sexually transmitted infections in China: multicenter cross‐sectional survey study. JMIR Public Health Surveill. 2024;10:e65713. 10.2196/65713 39761154 PMC11702827

[26] Auemaneekul N , Lertpruek S , Satitvipawee P , Tuah NA . Pre‐exposure prophylaxis uptake for HIV infection prevention among young men who have sex with men and transgender women in Bangkok, Thailand. J Health Res. 2020;35:434–443. 10.1108/jhr-10-2019-0242

[27] Bavinton B , Mahendra I , Law M , Kaldor J . Study report: Survei Kesehatan Seksual Indonesia 2018. Bali, Indonesia: Center for Public Health Innovation; 2019.

[28] Wu Y , Xie Lu , Meng S , Hou J , Fu R , Zheng H , et al. Mapping potential pre‐exposure prophylaxis users onto a motivational cascade: identifying targets to prepare for implementation in China. LGBT Health. 2019;6:250–260. 10.1089/lgbt.2018.0256 31170020 PMC6645195

[29] Ramautarsing RA , Meksena R , Sungsing T , Chinbunchorn T , Sangprasert T , Fungfoosri O , et al. Evaluation of a pre‐exposure prophylaxis programme for men who have sex with men and transgender women in Thailand: learning through the HIV prevention cascade lens. J Int AIDS Soc. 2020;23(Suppl 3):e25540. 10.1002/jia2.25540 32602660 PMC7325508

[30] Eger WH , Adaralegbe A , Khati A , Azwa I , Wickersham JA , Osborne S , et al. Exploring drivers of pre‐exposure prophylaxis uptake among gay, bisexual, and other men who have sex with men in Malaysia. Int J STD AIDS. 2022;33:821–828. 10.1177/09564624221106535 35772943 PMC10069270

[31] To KW , Lee SS . HIV pre‐exposure prophylaxis in South East Asia: a focused review on present situation. Int J Infect Dis. 2018;77:113–117. 10.1016/j.ijid.2018.10.027 30395980

[32] Thai Red Cross AIDS Research Centre . Consultation Report for the 3rd Asia‐Pacific Regional Consultation on PrEP Implementation. Bangkok: Thai Red Cross AIDS Research Centre; 2019.

[33] Nawawi F , Nugroho A , Wibowo IR . Breaking the stigma: increasing comprehensive HIV knowledge to end discrimination against people living with HIV. Int J Commun Occup Med. 2023;2:4. 10.53773/ijcom.v2i3.76.120-3

[34] Radics GB . Cultural wars and lesbian, gay, bisexual, transgender (LGBT) rights in Southeast Asia: ‘Asian values’, human rights, and the ‘homosexual turn’. Curr Sociol. 2024. 10.1177/00113921241241808

[35] Lau JY , Hung C‐T , Lee S‐S . A review of HIV pre‐exposure prophylaxis (PrEP) programmes by delivery models in the Asia‐Pacific through the healthcare accessibility framework. J Int AIDS Soc. 2020;23:e25531. 10.1002/jia2.25531 32603517 PMC7326464

[36] Schmidt H‐MA , McIver R , Houghton R , Selvey C , McNulty A , Varma R , et al. Nurse‐led pre‐exposure prophylaxis: a non‐traditional model to provide HIV prevention in a resource‐constrained, pragmatic clinical trial. Sex Health. 2018;15:595–597. 10.1071/SH18076 30257752

[37] WHO . Differentiated and simplified pre‐exposure prophylaxis for HIV prevention. Geneva: World Health Organization; 2022.

[38] Chan C , Fraser D , Vaccher S , Yeung B , Jin F , Amin J , et al. Overcoming barriers to HIV pre‐exposure prophylaxis (PrEP) coverage in Australia among Medicare‐ineligible people at risk of HIV: results from the MI‐EPIC clinical trial. Sex Health. 2022;18:453–459. 10.1071/SH21096 34895427

[39] van Dijk M , de Wit JBF , Guadamuz TE , Martinez JE , Jonas KJ . Slow uptake of PrEP: behavioral predictors and the influence of price on PrEP uptake among MSM with a high interest in PrEP. AIDS Behav. 2021;25:2382–2390. 10.1007/s10461-021-03200-4 33611697 PMC8222036

[40] Bavinton BR , Dharan NJ . Cost‐effectiveness of the expansion of PrEP for HIV infection in the Asia Pacific region. Lancet Glob Health. 2024;12:e177–e178. 10.1016/S2214-109X(23)00600-9 38245105

[41] Siregar AYM , Juwita MN , Hardiawan D , Akbar A , Rachman ZH , Haekal MDF , et al. Cost of implementing HIV pre‐exposure prophylaxis at community‐based clinics in Indonesia. Trop Med Int Health. 2024;29:13–22. 10.1111/tmi.13946 37926554

[42] Rivet Amico K , Bekker LG . Global PrEP roll‐out: recommendations for programmatic success. Lancet HIV. 2019;6:e137–e140. 10.1016/S2352-3018(19)30002-5 30660592

