# Correction to PrEP use and willingness cascades among GBMSM in 15 Asian countries/territories: An analysis of the PrEP APPEAL survey

**DOI:** 10.1002/jia2.26520

**Published:** 2025-06-10

Wirawan GBS, Schmidt HM, Chan C, Fraser D, Ong JJ, Cassell M, et al. PrEP use and willingness cascades among GBMSM in 15 Asian countries/territories: an analysis of the PrEP APPEAL survey. J Int AIDS Soc. 2025;28(1):e26438. https://doi.org/10.1002/jia2.26438

In the article, the middle initial of one of the authors was incorrect and should be changed to Kimberly E. Green from Kimberly A. Green.

The online version of the article was corrected.

We apologize for this error.

