# Supplementary material for: PrEP use and willingness cascades among GBMSM in 15 Asian countries/territories: an analysis of the PrEP APPEAL survey
Source: J Int AIDS Soc. 2025 Mar 28;28(4):e26438. doi: 10.1002/jia2.26438 (PMC11953173; doi:10.1002/jia2.26438)
Supplement: Supplementary file 1 — Table S1.1. PrEP use cascade by country. Table S1.2. PrEP willingness cascade by country. [file JIA2-28-e26438-s004.docx]

**Table S1.1. PrEP use cascade by country.**

| Country (n=13,899) | PrEP aware,  % (n/N) | Lifetime PrEP use, % (n/N) | Current PrEP Use, % (n/N) |
| --- | --- | --- | --- |
| Thailand (n=1,063) | 77.7 (826/1,063) | 42.3 (349/826) | 62.2 (217/349) |
| Vietnam (n=1,067) | 94.9 (1,012/1,067) | 71.6 (725/1,012) | 80.7 (585/725) |
| Indonesia (n=1,039) | 73.3 (762/1,039) | 14.3 (109/762) | 79.8 (87/109) |
| Philippines (n=1,420) | 75.7 (1,075/1,420) | 27.0 (290/1,075) | 79.7 (231/290) |
| China, exc. Hong Kong (n=1,543) | 92.0 (1,420/1,543) | 32.3 (458/1,420) | 63.1 (289/458) |
| Malaysia (n=712) | 87.2 (621/712) | 23.4 (145/621) | 64.1 (93/145) |
| Myanmar (n=382) | 91.6 (350/382) | 41.1 (144/350) | 75.7 (109/144) |
| India (n=1,304) | 51.7 (674/1,304) | 13.6 (92/674) | 55.4 (51/92) |
| Cambodia (n=472) | 79.2 (374/472) | 61.2 (229/472) | 62.5 (143/229) |
| Laos (n=236) | 49.6 (117/236) | 46.2 (54/117) | 44.4 (24/54) |
| Nepal (n=343) | 83.1 (285/343) | 78.6 (224/343) | 82.6 (185/224) |
| Taiwan (n=1,994) | 95.9 (1,912/1,994) | 31.6 (604/1,912) | 60.1 (363/604) |
| Singapore (n=607) | 92.8 (563/607) | 36.2 (204/563) | 71.1 (145/204) |
| Hong Kong (n=490) | 92.5 (453/490) | 30.7 (139/453) | 69.1 (96/139) |
| Japan (n=1,227) | 80.1 (983/1,227) | 23.8 (234/983) | 79.1 (185/234) |

**Table S1.2. PrEP willingness cascade by country.**

| Country (n=7,472) | Higher risk of HIV,  % (n/N) | Willing to use PrEP, % (n/N) | Willing to pay for PrEP, % (n/N) |
| --- | --- | --- | --- |
| Thailand (n=477) | 57.7 (275/477) | 71.6 (197/275) | 56.9 (112/197) |
| Vietnam (n=287) | 55.8 (160/287) | 74.4 (119/160) | 79.0 (94/119) |
| Indonesia (n=653) | 65.5 (428/653) | 79.0 (338/428) | 70.1 (237/338) |
| Philippines (n=785) | 46.0 (361/785) | 90.3 (326/361) | 69.6 (227/326) |
| China, exc. Hong Kong (n=962) | 50.8 (489/962) | 58.5 (286/489) | 90.2 (258/286) |
| Malaysia (n=476) | 52.3 (249/476) | 82.3 (205/249) | 93.2 (191/205) |
| Myanmar (n=206) | 54.9 (113/206) | 62.8 (71/113) | 56.3 (40/71) |
| India (n=582) | 44.3 (258/582) | 77.5 (200/258) | 79.0 (158/200) |
| Cambodia (n=145) | 60.0 (87/145) | 62.1 (54/87) | 40.7 (22/54) |
| Laos (n=63) | 77.8 (49/63) | 24.5 (12/49) | 66.7 (8/12) |
| Nepal (n=61) | 70.5 (43.61) | 32.6 (14/43) | 14.3 (2/14) |
| Taiwan (n=1,308) | 62.7 (820/1,308) | 73.8 (605/820) | 96.7 (585/605) |
| Singapore (n=359) | 41.2 (148/359) | 79.7 (118/148) | 98.3 (116/118) |
| Hong Kong (n=314) | 54.5 (171/314) | 67.8 (116/171) | 93.1 (108/116) |
| Japan (n=749) | 55.7 (417/749) | 80.3 (335/417) | 98.2 (329/335) |
