# Supplementary material for: PrEP use and willingness cascades among GBMSM in 15 Asian countries/territories: an analysis of the PrEP APPEAL survey
Source: J Int AIDS Soc. 2025 Mar 28;28(4):e26438. doi: 10.1002/jia2.26438 (PMC11953173; doi:10.1002/jia2.26438)
Supplement: Supplementary file 2 — Table S2. Subgroup analysis for reasons for stopping PrEP or not taking up PrEP based on being in a monogamous relationship as a reason for not being on PrEP. [file JIA2-28-e26438-s002.docx]

**Table S2. Subgroup analysis for reasons for stopping PrEP or not taking up PrEP based on being in a monogamous relationship as a reason for not being on PrEP.**

| Reasons | Stopped using PrEP (n=1,197) | | | Never tried PrEP n=2,996 | |
| --- | --- | --- | --- | --- | --- |
|  | I got into a monogamous relationship | | | | |
|  | No  (n=1,058) | Yes  (n=139) | No  (n=2,884) | | Yes  (n=112) |
| I am not having much sex | 445 (42%) | 51 (37%) | 572 (20%) | | 36 (32%) |
| Too expensive | 397 (29%) | 20 (14%) | 1,249 (43%) | | 37 (33%) |
| I’m concerned about the side effects | 287 (27%) | 23 (17%) | 946 (33%) | | 43 (38%) |
| I don’t like taking pills regularly | 214 (20%) | 26 (19%) | 415 (14%) | | 32 (29%) |
| I prefer to use condoms | 168 (16%) | 38 (27%) | 338 (12%) | | 31 (28%) |
| Not available where I live | `129 (12%) | 9 (7%) | 593 (17%) | | 14 (13%) |
| Don’t know where to get PrEP | 127 (12%) | 7 (5%) | 1,681 (58%) | | 46 (41%) |
| I am not at high risk of HIV | 110 (10%) | 21 (15%) | 144 (5%) | | 20 (18%) |
| Too inconvenient | 93 (9%) | 4 (3%) | 230 (8%) | | 8 (7%) |
| Can't get prescription | 87 (8%) | 8 (6%) | 538 (19%) | | 21 (19%) |
| COVID-19 made it too hard to get | 72 (7%) | 8 (6%) | 111 (4%) | | 7 (6%) |
| I’m concerned about what my friends and family would think of me | 47 (4%) | 8 (6%) | 344 (12%) | | 20 (18%) |
| My sexual partner(s) didn’t like me taking PrEP | 12 (1%) | 0 (0%) | 9 (<1%) | | 5 (5%) |
| I was denied access to PrEP by a healthcare provider | 9 (1%) | 2 (1%) | 33 (1%) | | 3 (3%) |
