# Supplementary material for: PrEP use and willingness cascades among GBMSM in 15 Asian countries/territories: an analysis of the PrEP APPEAL survey
Source: J Int AIDS Soc. 2025 Mar 28;28(4):e26438. doi: 10.1002/jia2.26438 (PMC11953173; doi:10.1002/jia2.26438)
Supplement: Supplementary file 3 — Table S3. Stratified logistic regression for factors associated with lifetime PrEP use. [file JIA2-28-e26438-s003.docx]

**Table S3. Stratified logistic regression for factors associated with lifetime PrEP use.**

| Variables | Countries with partial PrEP access^a^ (n=7,303) | | | | Countries with wider PrEP access^b^ (n=4,124) | | | |
| --- | --- | --- | --- | --- | --- | --- | --- | --- |
|  | OR (95% CI) | p-value | aOR (95% CI) | p-value | OR (95% CI) | p-value | aOR (95% CI) | p-value |
| Country groups  Lower-middle income  Upper-middle income  High income | Ref.  1.08 (0.96-1.22)  1.04 (0.92-1.18) | 0.459 |  |  | Ref.  0.33 (0.28-0.40)  0.21 (0.18-0.24) | <0.001^†^ | Ref.  0.28 (0.22-0.35)  0.16 (0.13-0.19) | <0.001* |
| Residence  Town, villages, or rural  Capital or large cities | Ref.  1.20 (1.08-1.34) | 0.001^†^ | Ref,  1.12 (0.98-1.28) | 0.090 | Ref.  0.98 (0.85-1.13) | 0.827 |  |  |
| Age groups  <20  20-29  30-39  40-49  50-59  ≥ 60 | Ref.  2.01 (1.40-2.90)  2.44 (1.69-3.52)  2.39 (1.63-3.50)  2.61 (1.72-3.97)  1.59 (0.76-3.32) | <0.001^†^ | Ref.  1.35 (0.89-2.07)  1.74 (1.14-2.68)  1.98 (1.27-3.10)  2.79 (1.71-4.55)  1.73 (0.75-3.96) | <0.001* | Ref.  1.74 (1.21-2.52)  1.48 (1.02-2.15)  1.12 (0.85-1.68)  0.88 (0.48-1.63)  0.66 (0.17-2.61) | <0.001^†^ | Ref.  1.89 (1.13-3.14)  2.01 (1.19-3.40)  2.00 (1.14-3.51)  2.25 (1.04-4.86)  1.12 (0.21-5.89) | <0.001* |
| Gender  Cisgender man/male  Not cisgender man/male | Ref.  1.95 (1.63-2.32) | <0.001^†^ | Ref.  1.84 (1.48-2.29) | <0.001* | Ref.  2.00 (1.60-2.49) | <0.001^†^ | Ref.  1.15 (0.86-1.53) | 0.346 |
| Sexual orientation  Gay  Not gay | Ref.  0.67 (0.59-0.75) | <0.001^†^ | Ref.  0.73 (0.64-0.84) | <0.001* | Ref.  0.98 (0.84-1.14) | 0.793 |  |  |
| Education levels  Without university degree  University degree | Ref.  0.79 (0.70-0.90) | <0.001^†^ | Ref.  0.98 (0.84-1.15) | 0.815 | Ref.  0.54 (0.46-0.63) | <0.001^†^ | Ref.  0.83 (0.66-1.03) | 0.091 |
| Employment  Not employed  Employed | Ref.  1.20 (1.07-1.35) | 0.003^†^ | Ref.  1.16 (1.00-1.35) | 0.058 | Ref.  1.16 (1.00-1.36) | 0.050^†^ | Ref.  1.24 (1.01-1.52) | 0.038* |
| Relationship  No  Yes | Ref.  1.33 (1.20-1.48) | <0.001^†^ | Ref.  1.17 (1.04-1.32) | 0.010* | Ref.  1.85 (1.63-2.09) | <0.001^†^ | Ref.  1.15 (0.98-1.35) | 0.081 |
| Social engagement in the LGBTQ+ community (2-10), each incremental score, median (IQR) | 1.17 (1.13-1.21) | <0.001^†^ | 0.99 (0.96-1.04) | 0.854 | 1.11 (1.06-1.15) | <0.001^†^ | 1.06 (1.01-1.11) | 0.027* |
| Sex work  Not in the last 6 mo  Sometimes in the last 6 mo  Sex work is primary income | Ref.  1.81 (1.51-2.18)  4.62 (3.42-6.22) | <0.001^†^ | Ref.  1.34 (1.07-1.66)  2.29 (1.58-3.33) | <0.001* | Ref.  1.74 (1.35-2.25)  5.42 (3.40-8.66) | <0.001^†^ | Ref.  1.12 (0.81-1.55)  1.29 (0.73-2.25) | 0.545 |
| Number of partners in last 6 months  0-1  2-5  > 5 | Ref.  1.92 (1.67-2.20)  4.64 (4.01-5.38) | <0.001^†^ | Ref.  1.36 (1.07-1.66)  2.29 (1.58-3.32) | <0.001* | Ref.  1.66 (1.43-1.94)  3.18 (2.67-3.78) | <0.001^†^ | Ref.  1.14 (0.94-1.38)  1.73 (1.39-2.17) | <0.001* |
| HIV status  HIV negative  Unknown | Ref.  0.21 (0.17-0.26) | <0.001^†^ | Ref.  0.77 (0.56-1.07) | 0.116 | Ref.  0.21 (0.17-0.26) | <0.001^†^ | Ref.  0.43 (0.30-0.61) | <0.001* |
| Last HIV test  In the last 12 months  Over 12 months ago  Never tested | Ref.  0.20 (0.17-0.24)  0.07 (0.05-0.10) | <0.001^†^ | Ref.  0.23 (0.19-0.27)  0.12 (0.08-0.19) | <0.001* | Ref.  0.22 (0.18-0.27)  0.05 (0.03-0.07) | <0.001^†^ | Ref.  0.28 (0.22-0.35)  0.09 (0.05-0.14) | <0.001* |
| Condomless sex in last 6 months  No  Yes | Ref.  2.54 (2.29-2.83) | <0.001^†^ | Ref.  1.70 (1.50-1.92) | <0.001* | Ref.  2.08 (1.83-2.36) | <0.001^†^ | Ref.  1.79 (1.51-2.11) | <0.001* |
| STI diagnosis in last 6 months  No  Yes | Ref.  2.94 (2.50-3.48) | <0.001^†^ | Ref.  1.51 (1.25-1.83) | <0.001* | Ref.  3.23 (2.59-4.05) | <0.001^†^ | Ref.  1.54 (1.17-2.01) | 0.002* |
| Chemsex in last 6 months  No  Yes | Ref.  3.11 (2.73-3.54) | <0.001^†^ | Ref.  1.88 (1.61-2.19) | <0.001* | Ref.  3.46 (3.00-4.24) | <0.001^†^ | Ref.  1.76 (1.46-2.12) | <0.001* |
| Injected drug use in last 6 months  No  Yes | Ref.  2.81 (2.27-3.47) | <0.001^†^ | Ref.  1.59 (1.21-2.07) | 0.001* | Ref.  2,24 (1.83-2.75) | <0.001^†^ | Ref.  1.48 (1.14-1.93) | 0.003* |
| Attitude: Willing to take PrEP to prevent HIV  Disagree  Agree and strongly agree | Ref.  1.70 (1.49-1.93) | <0.001^†^ | Ref.  1.52 (1.30-1.77) | <0.001* | Ref.  1.21 (1.04-1.40) | 0.012^†^ | Ref.  1.31 (1.07-1.59) | 0.008* |
| Attitude: Worried about PrEP side effects  Disagree  Agree and strongly agree | Ref.  0.61 (0.55-0.67) | <0.001^†^ | Ref.  0.60 (0.53-0.67) | <0.001* | Ref.  0.69 (0.61-0.78) | <0.001^†^ | Ref.  0.66 (0.56-0.76) | <0.001* |
| Attitude: Comfortable to discuss PrEP with Health Care Provider  Disagree  Agree and strongly agree | Ref.  1.72 (1.54-1.91) | <0.001^†^ | Ref.  1.47 (1.29-1.67) | <0.001* | Ref.  2.19 (1.91-2.50) | <0.001^†^ | Ref.  2.25 (1.89-2.69) | <0.001* |

^a^Partial PrEP access countries included Indonesia, Philippines, China, Malaysia, Myanmar, India, Lao PDR, Nepal, Singapore, Hong Kong (China), and Japan; ^b^Wider PrEP access countries included Thailand, Vietnam, Cambodia, and Taiwan; ^†^p<0.1, included in the multivariable model; *p<0.05, significant in the multivariable model
