# Supplementary material for: PrEP use and willingness cascades among GBMSM in 15 Asian countries/territories: an analysis of the PrEP APPEAL survey
Source: J Int AIDS Soc. 2025 Mar 28;28(4):e26438. doi: 10.1002/jia2.26438 (PMC11953173; doi:10.1002/jia2.26438)
Supplement: Supplementary file 4 — Table S4. Stratified logistic regression for factors associated with PrEP willingness among PrEP‐naïve participants with higher risk of HIV. [file JIA2-28-e26438-s001.docx]

**Table S4. Stratified logistic regression for factors associated with PrEP willingness among PrEP-naïve participants with higher risk of HIV.**

| Variables | Countries with partial PrEP access^a^ (n=2,726) | | | | Countries with wider PrEP access^b^ (n=1,342) | | | |
| --- | --- | --- | --- | --- | --- | --- | --- | --- |
|  | OR (95% CI) | p-value | aOR (95% CI) | p-value | OR (95% CI) | p-value | aOR (95% CI) | p-value |
| Country groups  Lower-middle income  Upper-middle income  High income | Ref.  0.60 (0.49-0.74)  1.03 (0.83-1.28) | <0.001^†^ | Ref.  0.64 (0.51-0.80)  1.16 (0.91-1.48) | <0.001* | Ref.  1.08 (0.74-1.58)  1.20 (0.88-1.64) | 0.471 |  |  |
| Residence  Town, villages, or rural  Capital or large cities | Ref.  0.97 (0.81-1.16) | 0.748 |  |  | Ref.  1.22 (0.93-1.60) | 0.156 |  |  |
| Age groups  <20  20-29  30-39  40-49  50-59  ≥ 60 | Ref.  0.77 (0.46-1.28)  0.70 (0.42-1.17)  0.69 (0.40-1.20)  0.72 (0.38-1.36)  0.28 (0.11-0.73) | 0.164 |  |  | Ref.  0.84 (0.45-1.57)  0.90 (0.48-1.70)  1.03 (0.51-2.09)  0.35 (0.13-0.92)  0.22 (0.03-1.43) | 0.104 |  |  |
| Gender  Cisgender man/male  Not cisgender man/male | Ref.  0.84 (0.60-1.17) | 0.304 |  |  | Ref.  0.67 (0.41-1.08) | 0.105 |  |  |
| Sexual orientation  Gay  Not gay | Ref.  1.03 (0.86-1.24) | 0.740 |  |  | Ref.  0.71 (0.53-0.95) | 0.021^†^ | Ref.  0.73 (0.54-1.00) | 0.052 |
| Education levels  Without university degree  University degree | Ref.  1.26 (1.02-1.54) | 0.031^†^ | Ref.  1.33 (1.07-1.68) | 0.012* | Ref.  1.52 (1.11-2.07) | 0.010^†^ | Ref.  1.21 (0.86-1.72) | 0.277 |
| Employment  Not employed  Employed | Ref.  1.01 (0.83-1.24) | 0.885 |  |  | Ref.  0.91 (0.67-1.23) | 0.556 |  |  |
| Relationship  No  Yes | Ref.  0.78 (0.65-0.92) | 0.005^†^ | Ref.  0.80 (0.66-0.97) | 0.020* | Ref.  0.94 (0.73-1.21) | 0.636 |  |  |
| Social engagement in the LGBTQ+ community (2-10), each incremental score, median (IQR) | 1.05 (1.00-1.11) | 0.073^†^ | 1.02 (0.96-1.08) | 0.548 | 1.06 (0.99-1.16) | 0.095^†^ | 0.99 (0.91-1.08) | 0.835 |
| Sex work  Not in the last 6 mo  Sometimes in the last 6 mo  Sex work is primary income | Ref.  2.69 (1.44-5.03)  1.92 (1.12-3.29) | 0.008^†^ | Ref.  2.74 (1.40-5.35)  2.24 (1.24-4.04) | 0.012* | Ref.  4.47 (1.55-12.91)  3.32 (1.30-8.49) | 0.021^†^ | Ref.  4.37 (1.40-13.63)  3.82 (1.37-10.67) | 0.031* |
| Number of partners in last 6 months  0-1  2-5  > 5 | Ref.  1.83 (1.49-2.23)  2.24 (1.75-2.86) | <0.001^†^ | Ref.  1.64 (1.32-2.04)  1.91 (1.45-2.51) | <0.001* | Ref.  2.60 (1.96-3.43)  3.17 (2.22-4.51) | <0.001^†^ | Ref.  2.46 (1.83-3.31)  3.26 (2.22-4.78) | <0.001* |
| HIV status  HIV negative  Unknown | Ref.  1.07 (0.86-1.34) | 0.525 |  |  | Ref.  0.99 (0.73-1.35) | 0.943 |  |  |
| Last HIV test  In the last 12 months  Over 12 months ago  Never tested | Ref.  1.06 (0.85-1.31)  1.27 (0.99-1.64) | 0.160 |  |  | Ref.  1.05 (0.77-1.43)  1.10 (0.78-1.55) | 0.842 |  |  |
| Condomless sex in last 6 months  No  Yes | Ref.  1.55 (1.22-1.96) | <0.001^†^ | Ref.  1.26 (0.93-1.70) | 0.130 | Ref.  1.13 (0.80-1.59) | 0.483 |  |  |
| STI diagnosis in last 6 months  No  Yes | Ref.  1.51 (1.12-2.04) | 0.005^†^ | Ref.  1.57 (1.13-2.17) | 0.007* | Ref.  1.31 (0.84-2.05) | 0.224 |  |  |
| Chemsex in last 6 months  No  Yes | Ref.  0.72 (0.59-0.88) | 0.002^†^ | Ref.  0.86 (0.68-1.10) | 0.242 | Ref.  0.86 (0.65-1.14) | 0.297 |  |  |
| Injected drug use in last 6 months  No  Yes | Ref.  0.60 (0.44-0.83) | 0.003^†^ | Ref.  0.64 (0.44-0.92) | 0.016* | Ref.  1.03 (0.72-1.50) | 0.846 |  |  |
| Attitude: Willing to take PrEP to prevent HIV  Disagree  Agree and strongly agree | Ref.  3.70 (3.07-4.47) | <0.001^†^ | Ref,  4.28 (3.47-5.28) | <0.001* | Ref.  3.43 (2.62-4.48) | <0.001^†^ | Ref.  3.26 (2.47-4.30) | <0.001* |
| Attitude: Worried about PrEP side effects  Disagree  Agree and strongly agree | Ref.  0.85 (0.71-1.02) | 0.072^†^ | Ref.  0.71 (0.58-0.86) | 0.001* | Ref.  0.93 (0.73-1.18) | 0.531 |  |  |
| Attitude: Comfortable to discuss PrEP with Health Care Provider  Disagree  Agree and strongly agree | Ref.  1.27 (1.07-1.51) | 0.006^†^ | Ref.  0.88 (0.72-1.08) | 0.223 | Ref.  1.09 (0.85-1.39) | 0.485 |  |  |

^a^Partial PrEP access countries included Indonesia, Philippines, China, Malaysia, Myanmar, India, Lao PDR, Nepal, Singapore, Hong Kong (China), and Japan; ^b^Wider PrEP access countries included Thailand, Vietnam, Cambodia, and Taiwan; ^†^p<0.1, included in the multivariable model; *p<0.05, significant in the multivariable model
